# A Flexible PVDF Sensor for Forcecardiography

**DOI:** 10.3390/s25051608

**Published:** 2025-03-06

**Authors:** Salvatore Parlato, Jessica Centracchio, Eliana Cinotti, Gaetano D. Gargiulo, Daniele Esposito, Paolo Bifulco, Emilio Andreozzi

**Affiliations:** 1 Department of Electrical Engineering and Information Technologies, University of Naples Federico II, Via Claudio, 21, 80125 Naples, Italy; salvatore.parlato@unina.it (S.P.); eliana.cinotti@unina.it (E.C.); emilio.andreozzi@unina.it (E.A.); 2 School of Engineering, Design and Built Environment, Western Sydney University, Penrith, NSW 2751, Australia; g.gargiulo@westernsydney.edu.au; 3 Department of Information and Electrical Engineering and Applied Mathematics, University of Salerno, Via Giovanni Paolo II, 132, 84084 Fisciano, Italy; daesposito@unisa.it

**Keywords:** forcecardiography, cardiac monitoring, respiratory monitoring, piezoelectric sensors, pvdf sensor, force sensors, seismocardiography, template matching, heart rate, respiratory rate

## Abstract

Forcecardiography (FCG) uses force sensors to record the mechanical vibrations induced on the chest wall by cardiac and respiratory activities. FCG is usually performed via piezoelectric lead-zirconate titanate (PZT) sensors, which simultaneously record the very slow respiratory movements of the chest, the slow infrasonic vibrations due to emptying and filling of heart chambers, the faster infrasonic vibrations due to movements of heart valves, which are usually recorded via Seismocardiography (SCG), and the audible vibrations corresponding to heart sounds, commonly recorded via Phonocardiography (PCG). However, PZT sensors are not flexible and do not adapt very well to the deformations of soft tissues on the chest. This study presents a flexible FCG sensor based on a piezoelectric polyvinylidene fluoride (PVDF) transducer. The PVDF FCG sensor was compared with a well-assessed PZT FCG sensor, as well as with an electro-resistive respiratory band (ERB), an accelerometric SCG sensor, and an electronic stethoscope for PCG. Simultaneous recordings were acquired with these sensors and an electrocardiography (ECG) monitor from a cohort of 35 healthy subjects (16 males and 19 females). The PVDF sensor signals were compared in terms of morphology with those acquired simultaneously via the PZT sensor, the SCG sensor and the electronic stethoscope. Moreover, the estimation accuracies of PVDF and PZT sensors for inter-beat intervals (IBIs) and inter-breath intervals (IBrIs) were assessed against reference ECG and ERB measurements. The results of statistical analyses confirmed that the PVDF sensor provides FCG signals with very high similarity to those acquired via PZT sensors (median cross-correlation index of 0.96 across all subjects) as well as with SCG and PCG signals (median cross-correlation indices of 0.85 and 0.80, respectively). Moreover, the PVDF sensor provides very accurate estimates of IBIs, with R^2^ > 0.99 and Bland–Altman limits of agreement (LoA) of [−5.30; 5.00] ms, and of IBrIs, with R^2^ > 0.96 and LoA of [−0.510; 0.513] s. The flexibility of the PVDF sensor makes it more comfortable and ideal for wearable applications. Unlike PZT, PVDF is lead-free, which increases safety and biocompatibility for prolonged skin contact.

## 1. Introduction

Recording heartbeat-induced mechanical vibrations provides valuable information about the mechanical activity of the heart to complement the electrical information provided by electrocardiography (ECG). Mechanical sensors attached onto the chest provide non-invasive diagnostic data on heart contraction, heart valves motion, and blood fluxes. Common examples include Phonocardiography (PCG) [1,2,3], Ballistocardiography (BCG) [4,5,6,7,8,9,10], Apexcardiography [11,12,13], Dynamocardiography [14], Kinetocardiography [15], and Seismocardiography (SCG) [16,17,18,19]. SCG records cardiac-induced vibrations of the chest wall via accelerometers and is currently one of the most popular techniques for prolonged, continuous cardiomechanical monitoring. One of the reasons for this is undoubtedly the availability of miniaturized accelerometers based on Micro Electromechanical Systems (MESMS) technologies, which has encouraged the development of pervasive continuous cardiac monitoring techniques, also via wearable systems [20,21]. Similarly to SCG, Gyrocardiography (GCG) uses MEMS gyroscopes to record rotational velocities of the chest surface, which provide similar information to SCG about cardiac mechanical activity [22,23]. Several inertial measurement unit (IMU) chips include both accelerometers and gyroscopes, so studies have been conducted to improve accuracy and performance using both signals [24,25,26,27]. As an example, Kinocardiography (KCG) uses the combination of two inertial units, placed on the chest and on the lower back, to provide additional information about the mechanical performance of the heart [28,29]. In addition to cardiac activity, IMUs can also be used to monitor respiratory activity. For example, by considering low-frequency changes in the components of the gravitational acceleration, a signal associated with respiratory motion of the ribcage can be obtained [30].

Forcecardiography (FCG) has recently emerged as a new technique for cardiac monitoring. It involves recording the pressure exerted by heart contractions onto the chest by means of force sensors [31]. The use of force sensors with a wide frequency response allows measurement of the large, slowly varying forces exerted by expansions and contractions of the ribcage during respiration, as well as the weak forces induced on the chest wall by the mechanical activity of the heart [31,32]. Indeed, the raw signal obtained from an FCG sensor contains a respiratory component, referred to as Forcerespirogram (FRG), a low-frequency infrasonic component (LF-FCG) related to movements of heart walls and blood volume changes during emptying and filling of heart chambers, a high-frequency infrasonic component (HF-FCG) corresponding to heart valves vibrations, and an audible component (HS-FCG) corresponding to heart sounds. FCG allows capturing all these components simultaneously from a single site on the chest, thus offering the ability to perform a multimodal cardio-respiratory monitoring for continuous long-term surveillance of healthy and pathological subjects [31].

The HF-FCG component shares a very similar waveform to the accelerometric SCG signal [33], while the HS-FCG component is very similar to PCG signals acquired via a common electronic stethoscope [31]. Hence, FCG offers much more information than SCG because accelerometers cannot capture very low-frequency movements generated by heart wall motion, nor audible vibrations (i.e., heart sounds) with adequate signal-to-noise ratio.

Until now, FCG devices have included a small piezoelectric lead–zirconate–titanate (PZT) sensor. However, the rigid structure of PZT sensors is poorly suited for embedding in garments for wearable applications. PZT ceramic is brittle and prone to cracking under mechanical stress, reducing its reliability in long-term applications. Furthermore, PZT sensors are subject to thermal drift, which can compromise output stability. A recent study reported that PZT contains up to 60% lead by weight, which is incorporated to enhance piezoelectric performance [34]. This high lead content could raise doubts about biocompatibility and suitability for prolonged skin contact.

To overcome these drawbacks, an FCG sensor was designed based on a flexible piezoelectric transducer made of polyvinylidene fluoride (PVDF). PVDF is a lead-free semi-crystalline polymer that offers a combination of flexibility, chemical resistance, and excellent piezoelectric properties. In addition, PVDF exhibits less pronounced pyroelectric properties with respect to PZT, making it less susceptible to thermal drift. PVDF sensors have been extensively studied and used in various medical applications due to their unique piezoelectric and flexible properties, which make them extremely suitable for patient monitoring. They are used in various applications, such as heart rate and respiration rate estimation and embedded in electronic stethoscopes and ultrasound transducers [35,36,37,38,39,40,41,42,43,44]. The flexibility of PVDF sensors allows them to be integrated into wearable devices, making continuous, long-term monitoring feasible [36,45]. PVDF sensitivity to mechanical changes and its durability make it an ideal choice for detecting subtle physiological mechanical signals.

The performance of the PVDF FCG sensor was assessed on a cohort of 35 healthy subjects. In particular, the FCG signals acquired via the PVDF sensor were compared with the FCG signals from a PZT sensor, the SCG signals from an accelerometer, the PCG signals from an electronic stethoscope, the respiratory signals from a respiratory band, and the ECG signals from an ECG monitor, acquired simultaneously. The results of statistical analyses confirmed that the PVDF sensor provides FCG signals with very high similarity to PZT FCG signals, SCG signals, and PCG signals, as well as comparable accuracy to the PZT sensor in the estimation of inter-beat and inter-breath intervals. The PVDF sensor is suitable for FCG studies, and, thanks to its flexibility, it could be more easily integrated into wearable devices for continuous, long-term cardio-respiratory monitoring.

## 2. Materials and Methods

### 2.1. Development of the PVDF Forcecardiography Sensor

**Figure 1:**
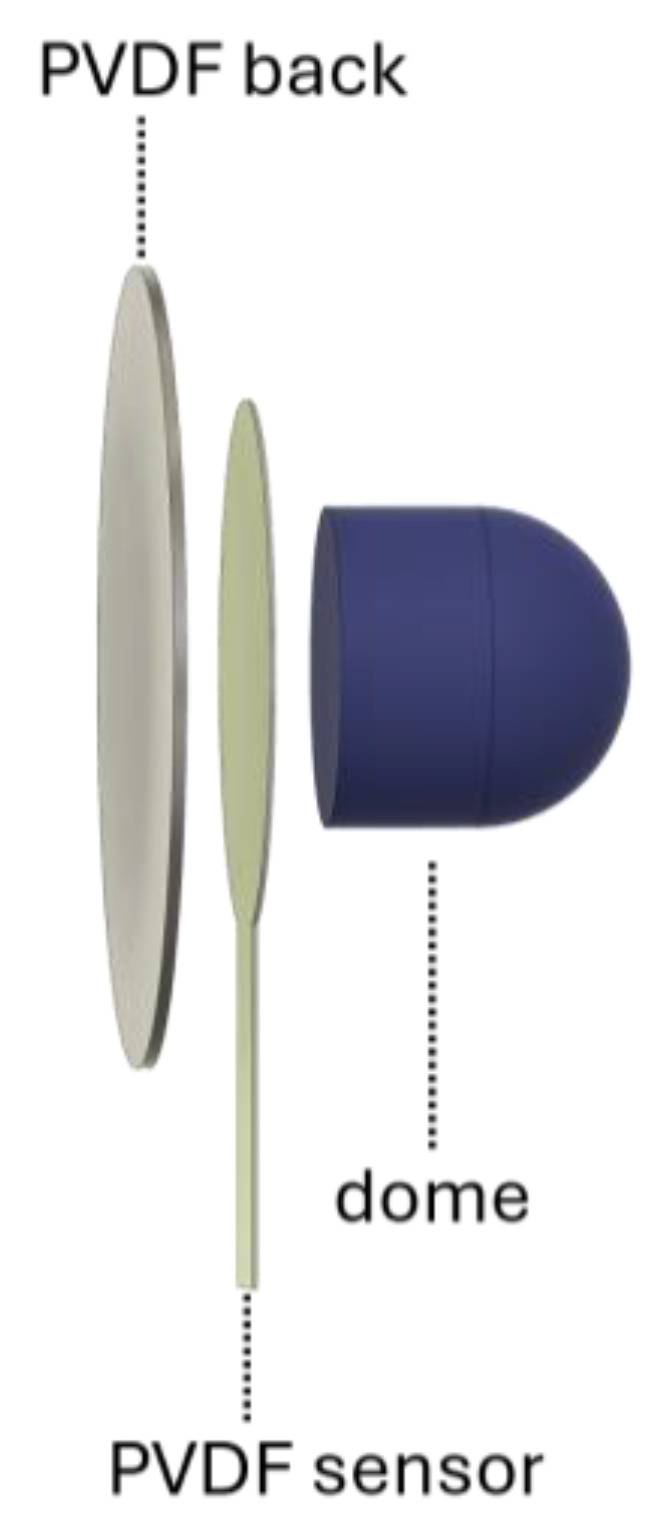
A 3D rendering of the flexible PVDF FCG sensor.

In this study, a commercial off-the-shelf PVDF piezoelectric transducer (PolyK Technologies, LLC, State College, PA, USA) was used to capture FCG signals. The transducer consisted of a 110 µm thick PVDF film with screen-printed thick Ag electrodes and had a diameter of 20 mm and a capacitance of 340 pF, measured via an LCR meter (LCR-816, Good Will Instrument Co., Ltd., New Taipei City, Taiwan). The PVDF FCG sensor was realized by encasing the PVDF transducer into a 3D-printed thermoplastic polyurethane (TPU) holder and attaching a silicone dome structure to the transducer bottom as a mechanical coupler to stabilize skin contact (see Figure 1). The same circuit described in [31] was used as a conditioning circuit for the PDVF FCG sensor to ensure that the low-frequency respiratory components were preserved.

The performance of the PVDF FCG sensor was assessed via different analyses. First, a morphological comparison was carried out between the FCG signals acquired by the PVDF sensor and the PZT sensor to confirm the suitability for FCG studies. Additional morphological comparisons were performed between HF-FCG components of PVDF sensor signals and SCG signals from an accelerometer, as well as between HS-FCG components and PCG signals from an electronic stethoscope. Further investigations were conducted to assess the performance of the PVDF FCG sensor in cardio-respiratory monitoring. To this aim, a sensors assembly, including PVDF, PZT, and accelerometer sensors, was developed, as well as a proper measurement setup comprising an electronic stethoscope, a respiratory band, and an ECG monitor.

### 2.2. Sensors Assembly for Simultaneous Measurements

Signals acquired via the PVDF sensor were compared with those provided by a PZT sensor similar to the one used in [31] and an ADXL-335 accelerometer (Analog Devices, Inc., One Analog Way, Wilmington, MA, USA). The PZT sensor used in this study had an external diameter of 20 mm, and an electrical capacitance of 22 nF measured via the LCR meter (LCR-816, Good Will Instrument Co., Ltd., New Taipei City, Taiwan).

**Figure 2:**
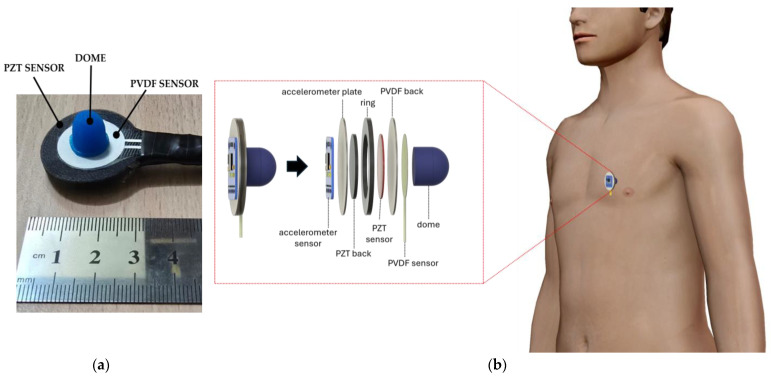
(**a**) Actual view of the sensors assembly; (**b**) 3D rendering of sensors assembly with an exploded view of its components and a typical placement onto a subject’s chest.

To acquire simultaneous FCG and SCG measurements from the same site on the chest, an ad hoc sensors assembly was designed (see Figure 2). In particular, the PZT sensor was fitted inside a TPU ring with a matching inner diameter, and its back was shielded via a protective TPU back cover. The total thickness of the PZT sensor and its TPU back cover matched the whole TPU ring thickness. The PZT sensor with its TPU enclosure was attached to the back of the PVDF sensor, equipped with the dome-shaped mechanical coupler. The flexible behavior of the PVDF sensor allowed the dome to act simultaneously as a mechanical coupler also for the PZT sensor, as in [31]. Finally, the accelerometer was attached to the FCG sensors. To this purpose, the accelerometer was first fixed to a TPU plate, which was then attached to the PZT back.

Figure 2a shows a picture of the sensors assembly, while Figure 2b shows a 3D rendering of the sensors assembly with an exploded view of its components and a typical positioning onto a subject’s chest.

### 2.3. Measurement Setup and Protocol

Thirty-five healthy subjects (16 males and 19 females, aged 26.3 ± 5.13 years) were involved in the experimental tests for the performance assessment of the PVDF FCG sensor. This study received the approval by the “Ethics Committee for research with human subjects” of the University of Naples Federico II (approval n.: PG/2024/0026606).

**Figure 3:**
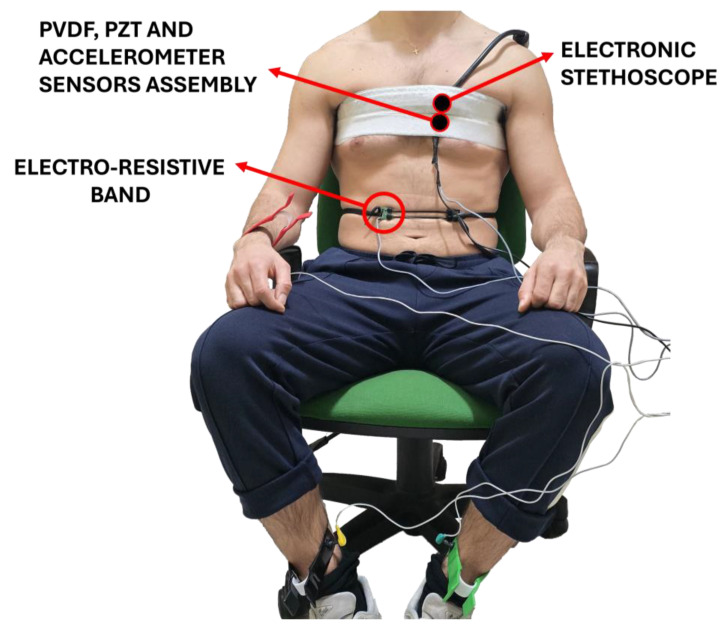
An example of a subject equipped with the measurement setup.

The sensors assembly was placed onto the subjects’ chests and secured via medical adhesive tape. In particular, it was positioned on the fourth intercostal space by locating the point of maximal signal amplitude for each subject. For comparison purposes, an ECG lead II was acquired by means of a WelchAllyn Propaq^®^ Encore monitor (Welch Allyn Inc., New York, NY, USA) and used as reference to evaluate the performance of FCG sensors in heart monitoring. Furthermore, simultaneous records were carried out with an Aethra Telestethphone electronic stethoscope to record reference PCG signals. Specifically, the electronic stethoscope was placed as close as possible to the sensor assembly, and both were fastened to the subjects’ chests via an elastic belt (see Figure 3).

The respiration monitoring technique described by [46], previously used in [32], was chosen as the reference method to evaluate the performance of the PVDF sensor in respiration monitoring. This involves an electro-resistive band (ERB), which is a stretchable strip made of conductive rubber that exhibits increased electrical resistance when stretched. As a result, it effectively tracks the expansion and contraction of the belly during the inhalation and exhalation phases. The ERB was secured around subjects’ torsos at the abdomen. An example of a subject equipped with the measurement setup is depicted in Figure 3.

A five-minute recording was acquired for each of the 35 subjects involved in the study, during quiet breathing while sitting comfortably on a chair, keeping their back straight against the seatback. FCG signals from the flexible PVDF sensor and the PZT sensor, SCG signals from the accelerometer, PCG signals from the electronic stethoscope, respiratory signals from the ERB, and ECG lead II signals were all synchronously recorded via a National Instrument NI-USB6212 DAQ board (National Instruments Corp., Austin, TX, USA) with a sampling frequency of 10 kHz and 16-bit precision.

### 2.4. Signals Pre-Processing

MATLAB^®^ R2023b (The MathWorks, Inc., 1 Apple Hill Drive, Natick, MA, USA) was used for signal processing and analysis. ECG signals were band-pass filtered in the 0.5–40 Hz band using a 4th-order zero-lag Butterworth filter. A comb notch filter was then applied to remove 50 Hz powerline interference and its higher harmonics. Finally, R-peaks were detected using the well-known Pan and Tompkins algorithm [47], implemented via the BioSigKit Matlab^®^ toolbox [48]. On the other hand, SCG and PCG signals were processed via a 4th-order zero-lag Butterworth filter with bandwidths of 7–30 Hz and 30–200 Hz, respectively.

**Figure 4:**
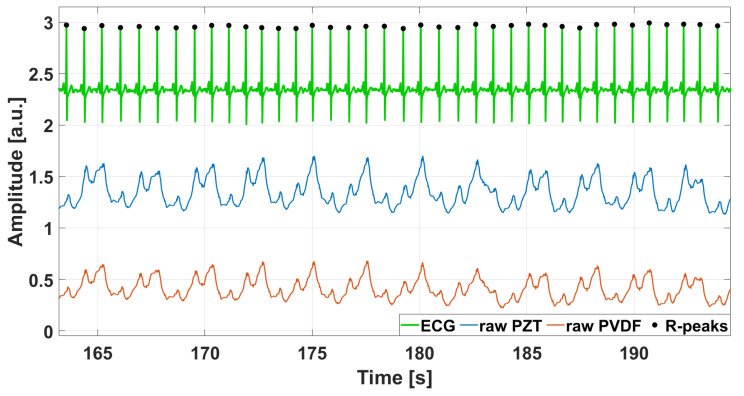
Excerpt of FCG sensors signals acquired from subject #3, displayed together with the reference ECG signal. Raw signals from PZT sensor and PVDF sensor were depicted with blue and red lines, respectively. Black dots mark the R-peaks detected on the ECG signal.

**Figure 5:**
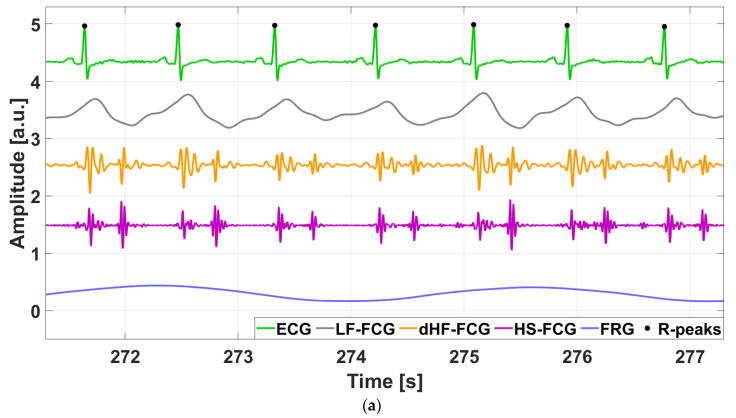

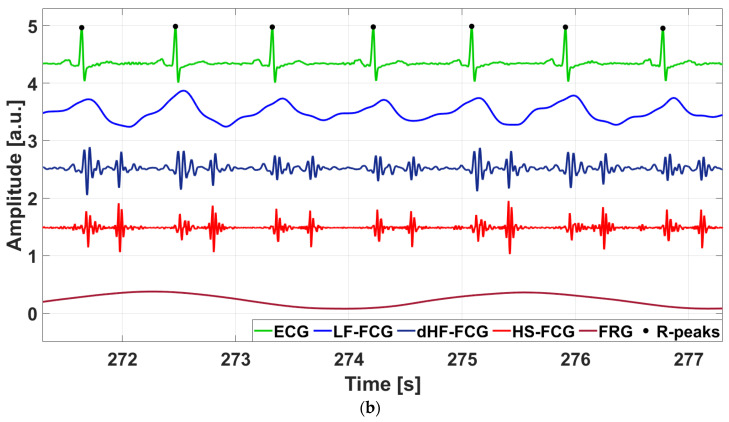
Excerpt of cardiac components extracted from FCG signals, with FRG, and ECG signals from subject #3: (**a**) PZT sensor signals; (**b**) PVDF sensor signals.

The raw signals captured by PZT and PVDF sensors featured a large component related to respiration (FRG) and a small, superimposed component related to cardiac activity. A Savitzky–Golay filter [49] with an order of 21 and a frame length ranging from 8 to 18 s was used to extract the FRG component, which was then subtracted from the raw signal to isolate the actual cardiac component (FCG). This filtering operation was essential to preserve the true shape of the FRG component and to prevent artifacts on the FCG signal. Afterward, the actual FCG signals were divided into the three cardiac components, namely LF-FCG, HF-FCG, and HS-FCG, using 4th-order Butterworth band-pass filters with frequency bands set to 0.5–6 Hz, 7–30 Hz, and 30–200 Hz, respectively. The first derivative of HF-FCG (dHF-FCG) component was then computed. Figure 4 shows an example of the raw signals acquired simultaneously from the PZT and PVDF sensors, together with the ECG signal. It can be observed that both FCG signals have a large respiratory component with a smaller superimposed cardiac component. Figure 5 shows all components extracted from FCG signals were provided by PZT and PVDF sensors, along with the ECG signal.

Savitzky–Golay filtering was applied to the raw ERB signals by using the same parameters adopted for the corresponding raw FCG signals.

### 2.5. Morphological Comparisons

The ECG-triggered ensemble averages (synchronized with R-peaks) of the PVDF FCG signals were compared with those of the PZT FCG signals to evaluate the suitability of the flexible PVDF sensor for FCG measurements. The time lags between the PVDF and PZT ensemble averages were determined by locating the absolute maximum of their normalized cross-correlation (NCC) functions. The PVDF and PZT ensemble averages were re-aligned based on their time lags, and then their Pearson’s correlation coefficients were computed to assess their similarity. The same procedure was adopted to quantify the similarity between the dHF-FCG signals provided by the PVDF sensor and the SCG signals acquired via the accelerometer, and the similarity between the HS-FCG signals provided by the PVDF sensor and the PCG signals acquired via the electronic stethoscope.

### 2.6. Cardiac Monitoring Performance

The cardiac monitoring performances of the flexible PVDF sensor and the PZT sensor were assessed by evaluating the accuracy of heartbeat detection and of inter-beat intervals estimation against a reference simultaneous ECG signal. The performance of the two FCG sensors were compared. To this aim, the dHF-FCG and HS-FCG components of PVDF and PZT sensors signals were considered.

**Figure 6:**
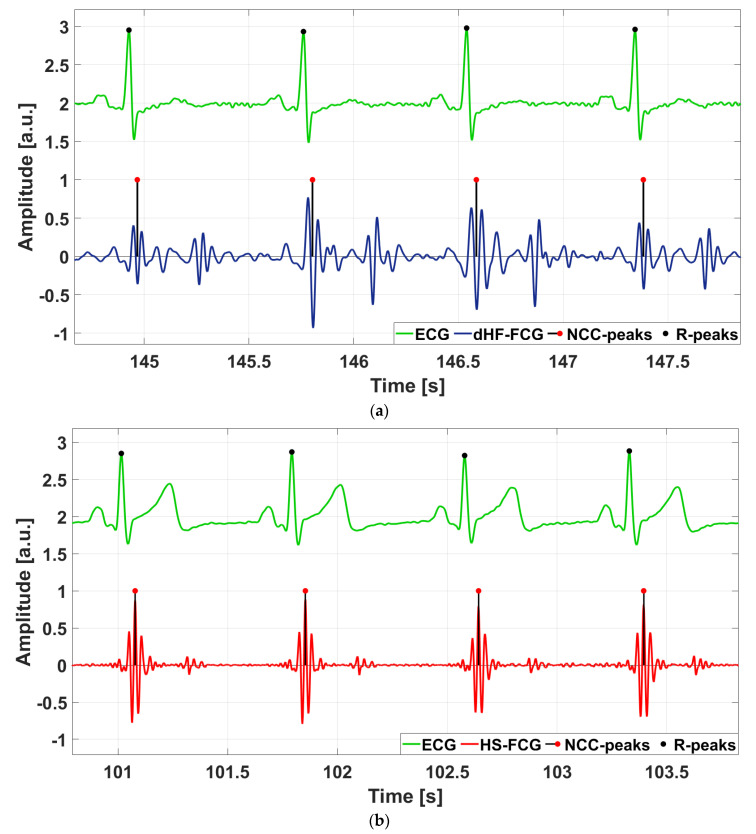
Heartbeats detection on dHF-FCG and HS-FCG, compared with the reference ECG signal: (**a**) heartbeats localization on dHF-FCG from subject #3; (**b**) heartbeats localization on HS-FCG from subject #32.

Heartbeat detection was performed via a template matching approach, already used in FCG [50,51], SCG, GCG [52,53,54,55], and sphygmic wave signals [36]. This approach requires a manual selection of a heartbeat template from the signal to be analyzed and localizes potential matches in the signal by using the NCC function as a similarity measure. To this aim, the NCC function is computed between the template and the whole signal and local maxima (NCC-peaks), corresponding to signal chunks that most resemble the heartbeat template are identified to obtain heartbeats locations. NCC-peaks were detected via the MATLAB^®^ function *findpeaks*, setting a minimum peak prominence of 0.5 and a minimum peak distance of 500 ms for each subject. An example of heartbeats localization is depicted in Figure 6.

After heartbeats localization, correct heartbeat detections (true positives, TPs), false heartbeat detections (false positives, FPs) and missed heartbeats (false negatives, FNs) were annotated with the support of reference ECG signals, as in [52,53]. Finally, inter-beat intervals (IBIs) were estimated from ECG, dHF-FCG, and HS-FCG signals as temporal differences between consecutive heartbeats locations.

### 2.7. Respiratory Monitoring Performance

The respiratory monitoring performances of the flexible PVDF sensor and the PZT sensor were assessed by evaluating the accuracy of respiratory acts detection and of inter-breath intervals (IBrIs) estimation against a reference simultaneous respiratory signal provided by the ERB. The performance of the PVDF and PZT sensors were compared.

**Figure 7:**
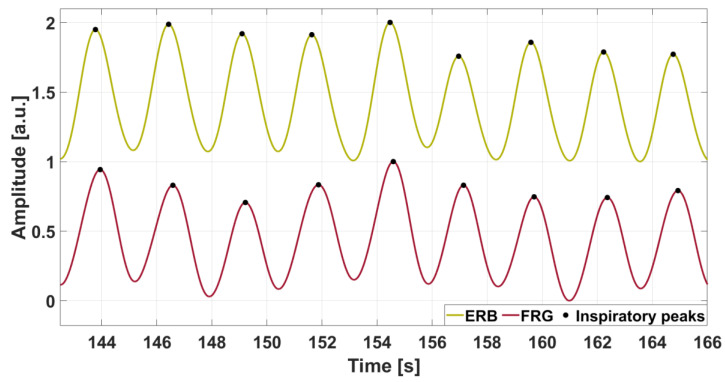
Comparison between the FRG extracted from the PVDF sensor signal from subject #3 and the reference ERB signal. The respiratory acts are marked with black dots.

Figure 7 shows an FRG signal from a subject provided by the PVDF sensor in comparison with the reference ERB signal, with respiratory acts also identified. A very slight time lag can be observed between the two respiratory signals. The delay may vary depending on the individual’s breathing pattern. In diaphragmatic (or abdominal) breathing, the diaphragm contracts and pushes down, causing the abdomen to expand first, while chest movement is minimal or occurs with a slight delay. In thoracic (or chest) breathing, the intercostal muscles drive the expansion of the chest more clearly, and the delay between the two signals can be reduced because the chest and abdomen move more synchronously. The specific breathing style and contribution of these muscle groups influence the delay observed between the two signals.

Respiratory acts were detected both in FRG and ERB signals via the MATLAB^®^ function *findpeaks* by appropriately setting the minimum peak prominence and minimum peak distance for each subject. The TPs, FPs, and FNs of respiratory acts detection were annotated for FRG signals acquired by the PVDF sensor and the PZT sensor with the support of the reference ERB signals. Finally, inter-breath intervals were estimated from ERB and FRG signals as temporal differences between consecutive respiratory acts.

### 2.8. Statistical Analyses

Sensitivity and positive predictive value (PPV) were used as metrics to assess the performances of the flexible PVDF sensor in the detection of heartbeats and respiratory acts and compare it with the performances of the PZT sensor. To evaluate the estimation accuracy of instantaneous heart and respiration rates, the inter-beat intervals obtained from dHF-FCG and HS-FCG of PVDF and PZT sensors were individually compared with those provided by the reference ECG signal, and the inter-breath intervals obtained from the FRG signals were compared with those provided by the reference ERB signal. Comparisons were performed via statistical analyses, specifically linear regression, correlation, and Bland–Altman analyses [56,57], implemented in the MATLAB^®^ function *bland–altman-and-correlation-plot* [58]. Inter-beat intervals and inter-breath intervals corrupted by FPs and FNs were excluded from the analyses.

## 3. Results

### 3.1. Morphological Comparisons

#### 3.1.1. Comparison of FCG Signals from PVDF and PZT Sensors

**Figure 8:**
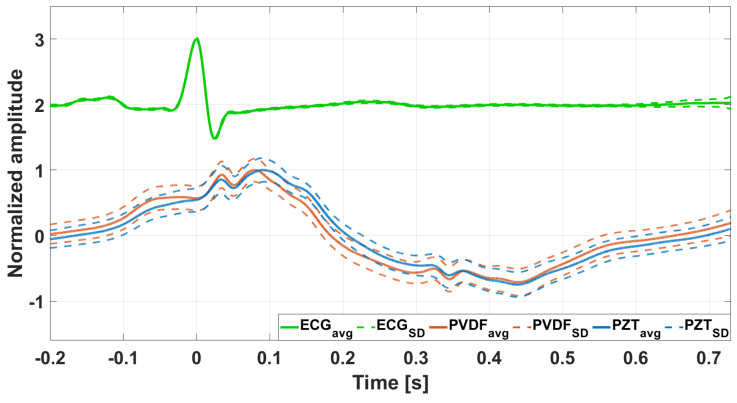
Illustration of the ECG-triggered ensemble averages (computed on approximately 230 heartbeats) of the raw FCG signals provided by the two sensors, along with ECG ensemble averages and limits of the ±SD. The ensemble averages are depicted with solid lines, while the limits of ±SD are depicted with dotted lines and normalized to the maximum of the ensemble average.

Figure 8 shows an example of ECG-triggered ensemble averages (synchronized with R-peaks and computed on about 230 heartbeats) of the FCG signals and the related ECG signal from subject #3, depicted as continuous lines. Their standard deviation (SD) ranges, which provide a measure of morphological variability, are also shown as dashed lines. It is possible to observe that the PVDF sensor signal and the PZT sensor signal exhibit very narrow SD ranges. This minimal variability in the shape of the FCG signal among multiple heartbeats demonstrates that also the PVDF sensor is capable of accurate and reproducible FCG measurements.

The morphological comparison between the ensemble averages of FCG signals from PVDF and PZT sensors reported Pearson’s correlation coefficients that were all statistically significant (*p* < 0.05), with a median value of 0.96 and an inter-quartile range (IQR) of [0.92, 0.98]. The correlation coefficients obtained for each subject are outlined in Table S1 in the Supplementary Materials. In addition, the analysis showed that the FCG signals from the PZT sensor were delayed, on average, by about 7 ms. This strong similarity confirmed the suitability of the flexible PVDF sensor for FCG applications.

#### 3.1.2. Comparison with SCG and PCG Signals

**Figure 9:**
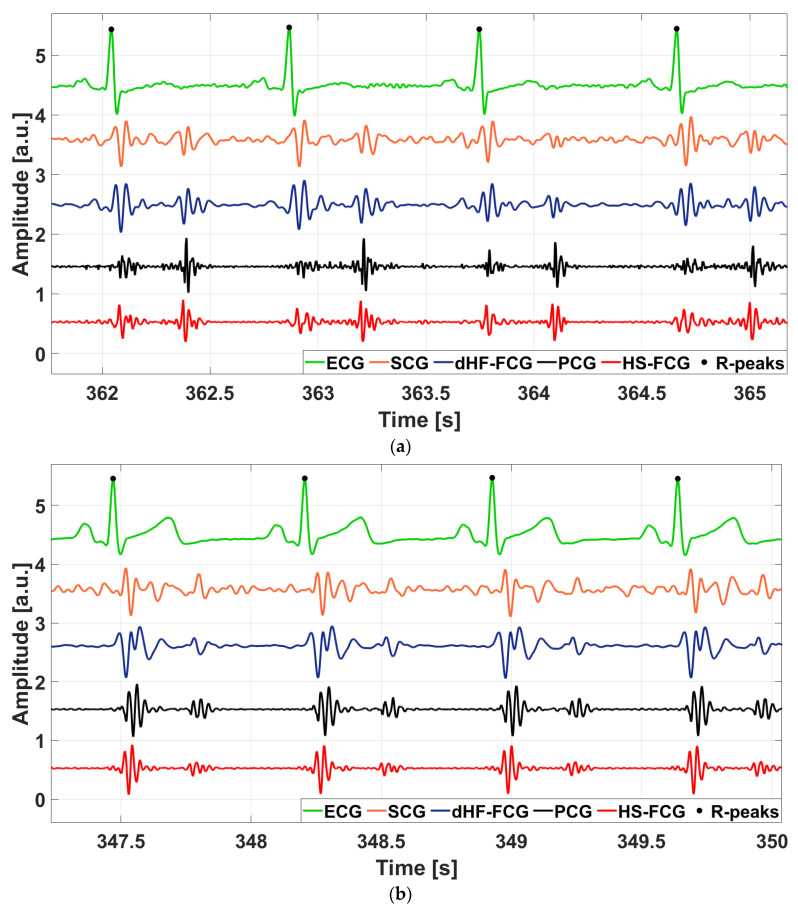
Excerpts of ECG, SCG, PCG, and FCG signals acquired simultaneously from two subjects involved in the experiment: (**a**) ECG, SCG, PCG, dHF-FCG, and HS-FCG of subject #3; (**b**) ECG, SCG, PCG, dHF-FCG, and HS-FCG of subject #32.

**Figure 10:**
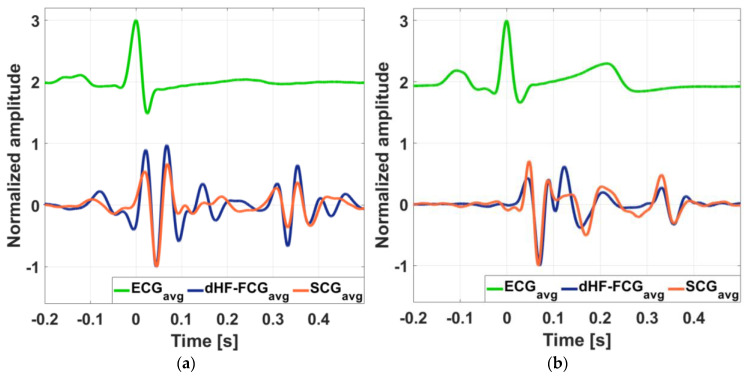
Comparison of aligned ECG-triggered ensemble averages of SCG (orange line) and dHF-FCG (dark blue line) signals, along with the ensemble average of the ECG (green line): (**a**) aligned ensemble averages of SCG and dHF-FCG signals from subject #3; (**b**) aligned ensemble averages of SCG and dHF-FCG signals from subject #32.

**Figure 11:**
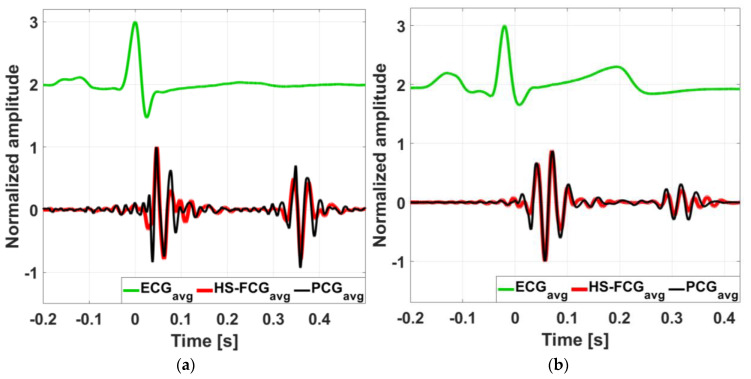
Comparison of aligned ECG-triggered ensemble averages of PCG (black line) and HS-FCG (red line) signals, along with the ensemble average of the ECG (green line): (**a**) aligned ensemble averages of PCG and HS-FCG signals from subject #3; (**b**) aligned ensemble averages of PCG and HS-FCG signals from subject #32.

Figure 9 shows an example of dHF-FCG and HS-FCG signals acquired by the PVDF sensor on two subjects, along with the related SCG and PCG signals acquired simultaneously. ECG signals are also shown as heartbeat references. It can be noted, by visual inspection, that accelerometric SCG signals exhibit a higher noise floor with respect to the related dHF-FCG signals acquired by the PVDF sensor. Figure 10 shows some examples of the aligned ensemble averages of dHF-FCG and SCG signals, along with the ensemble average of the ECG signal of subjects #3 and #32. Figure 11, on the other hand, shows an example of the aligned ensemble averages of the HS-FCG and PCG signals, along with that of the ECG signal of subjects #3 and #32.

The morphological comparison of the ensemble averages of PVDF dHF-FCG and HS-FCG signals with, respectively, SCG and PCG signals, reported Pearson’s correlation coefficients that were all statistically significant (*p* < 0.05) with a median value of 0.85 and an IQR of [0.80, 0.90] for dHF-FCG vs. SCG signals, and a median value of 0.80 and an IQR of [0.74, 0.83] for HS-FCG vs. PCG signals. The results achieved for each subject are reported in Table S1 in the Supplementary Materials. The ensemble averages of SCG signals were found to be delayed with respect to the related dHF-FCG signals by an average time lag of about 1 ms, while for the ensemble averages of PCG signals, an average time lag of about 11 ms was found with respect the related HS-FCG signals.

### 3.2. Heart Monitoring

#### 3.2.1. PVDF Sensor Performance

**Figure 12:**
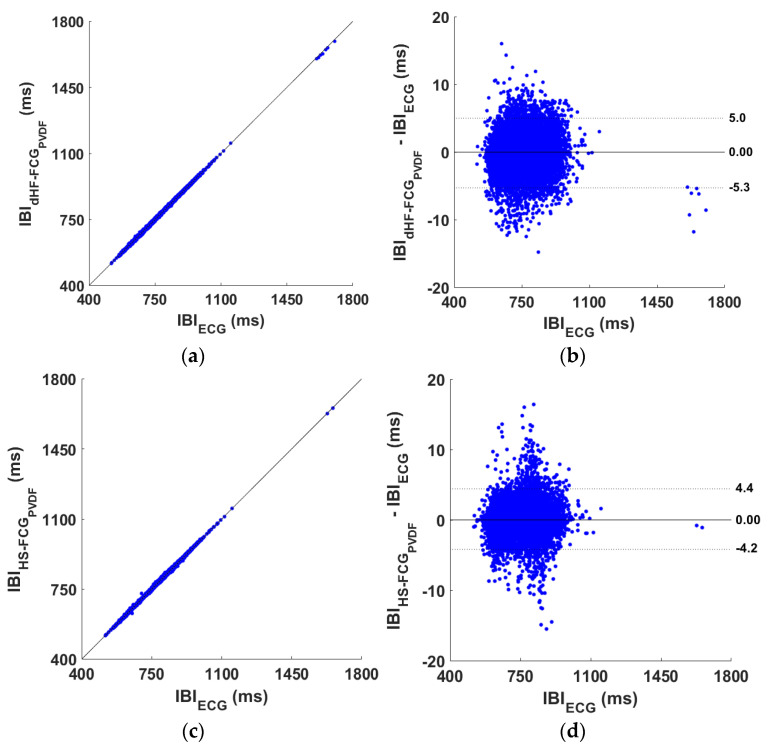
Statistical analyses on the inter-beat intervals (IBIs) obtained from dHF-FCG, HS-FCG (extracted from the PVDF sensor signal), and reference ECG signals: (**a**) results of regression and correlation analysis achieved from dHF-FCG signal; (**b**) results of Bland–Altman analysis achieved from dHF-FCG signal; (**c**) results of regression and correlation analysis achieved from HS-FCG signal; (**d**) results of Bland–Altman analysis achieved from HS-FCG signal.

The heartbeat detection performances of the dHF-FCG and HS-FCG signals acquired by the PVDF FCG sensor are outlined, respectively, in Tables S2 and S3 in the Supplementary Materials. The tables report, for each subject, the number of heartbeats correctly detected (true positives, TPs), as well as the number of FPs, FNs, and IBIs considered for statistical analyses. Overall, 14,171 and 11,851 heartbeats were correctly identified, respectively, in dHF-FCG and HS-FCG signals, out of a total of 15,251 reference heartbeats. A sensitivity and PPV of 92.9% and 96.9%, and of 77.7% and 86.8% were achieved, respectively, for dHF-FCG and HS-FCG signals (see Table 1). The results of regression and correlation analyses performed on the IBIs estimated from dHF-FCG and HS-FCG signals are outlined in Table 2, while the results of the Bland–Altman analyses are reported in Table 3. The regression and Bland–Altman plots are shown in Figure 12. The statistical analyses, carried out on a total of 13,412 IBIs estimated from the dHF-FCG and reference ECG signals, reported a coefficient of determination (R^2^) in excess of 0.99, a unit slope, and intercept of −0.938 ms, as well as a non-significant bias and limits of agreement (LoA) of [−5.300; 5.000] ms. On the other hand, the statistical analyses performed on a total of 10023 IBIs, estimated from the HS-FCG and reference ECG signals, reported an R^2^ in excess of 0.99, a unit slope, an intercept of −1.273 ms, along with a non-significant bias and LoAs of [−4.200; 4.400] ms.

#### 3.2.2. PZT Sensor Performance

**Figure 13:**
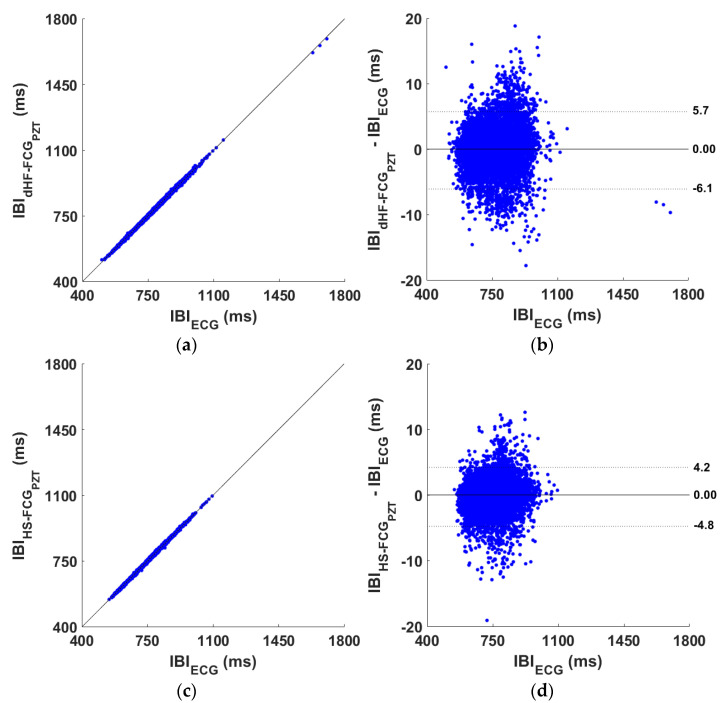
Statistical analyses on the inter-beat intervals (IBIs) obtained from dHF-FCG, HS-FCG (extracted from the PZT sensor signal) and reference ECG signals: (**a**) results of regression and correlation analysis achieved from dHF-FCG signal; (**b**) results of Bland–Altman analysis achieved from dHF-FCG signal; (**c**) results of regression and correlation analysis achieved from HS-FCG signal; (**d**) results of Bland–Altman analysis achieved from HS-FCG signal.

The heartbeat detection performances of the dHF-FCG and HS-FCG signals acquired by the PZT sensor are outlined, respectively, in Tables S4 and S5 in the Supplementary Materials. The tables report, for each subject, the number of TPs, FPs, FNs, and IBIs for statistical analyses. Overall, 14,209 and 11,439 heartbeats were correctly identified, respectively, in dHF-FCG HS-FCG signals, out of a total of 15,251 reference heartbeats. A sensitivity and PPV of 93.2% and 98.2%, and of 75.0% and 88.8% were achieved, respectively, for dHF-FCG and HS-FCG signals (see Table 1). The results of regression and correlation analyses performed on the IBIs estimated from dHF-FCG and HS-FCG signals are outlined in Table 2, while the results of the Bland–Altman analyses are reported in Table 3. The regression and Bland–Altman plots are shown in Figure 13. The statistical analyses carried out on a total of 13495 IBIs estimated from the dHF-FCG and ECG signals reported an R^2^ in excess of 0.99, a unit slope, and an intercept of −1.847 ms, as well as a non-significant bias and LoAs of [−6.100; 5.700] ms. On the other hand, the statistical analyses performed on a total of 9519 inter-beat intervals estimated from the HS-FCG and ECG signals report an R^2^ in excess of 0.99, a unit slope, and intercept of −1.743 ms, along with a non-significant bias and LoAs of [−4.800; 4.200] ms.

### 3.3. Respiration Monitoring

#### 3.3.1. PVDF Sensor Performance

**Figure 14:**
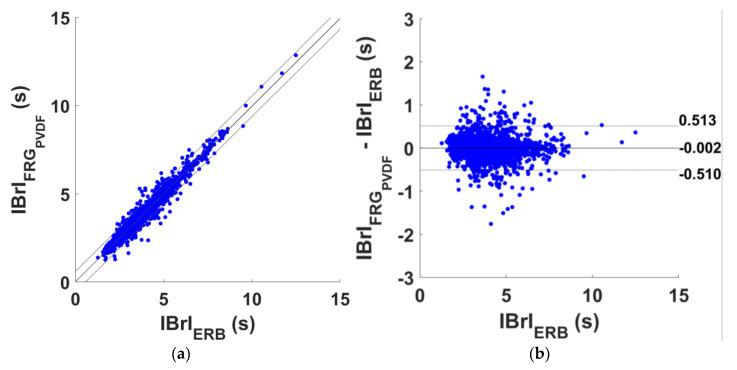
Statistical analyses on the inter-breath intervals (IBrIs) obtained from ERB and FRG signal provided by PVDF sensor signals: (**a**) results of regression and correlation analyses; (**b**) results of Bland–Altman analysis.

The respiratory acts detection performances of the FRG signals acquired by the PVDF sensor are reported in Table S6 in the Supplementary Materials, which outlines, for each subject, the number of TPs, FPs, FNs, and IBrIs considered for statistical analyses. Overall, 2895 respiratory acts were correctly identified out of a total of 3045 respiratory acts identified in the reference ERB signals, which yielded a sensitivity of 95.1% and a PPV of 92.5% (see Table 4). The results of regression and correlation analyses performed on the IBrIs estimated from FRG signals are outlined in Table 5, while the results of the Bland–Altman analyses are reported in Table 6. The regression and Bland–Altman plots are shown in Figure 14. The statistical analyses carried out on a total of 2752 IBrIs reported an R^2^ in excess of 0.96, a unit slope, and an intercept of 0.019 s, as well as a non-significant bias and LoAs of [−0.510; 0.513] ms.

#### 3.3.2. PZT Sensor Performance

**Figure 15:**
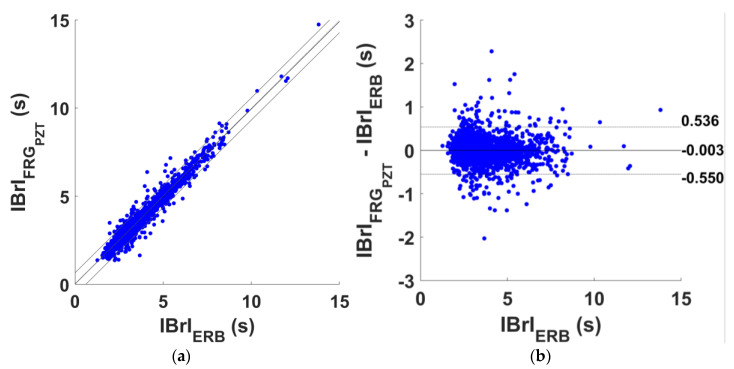
Statistical analyses on the inter-breath intervals (IBrIs) obtained from ERB and FRG signal provided by PZT sensor signals: (**a**) results of regression and correlation analyses; (**b**) results of Bland–Altman analysis.

The respiratory acts detection performances of the FRG signals acquired by the PVDF sensor are reported in Table S7 in the Supplementary Materials, which outlines, for each subject, the number of TPs, FPs, FNs, and IBrIs considered for statistical analyses. Overall, 2868 respiratory acts were correctly identified out of a total of 3045 respiratory acts identified in the reference ERB signals, which yielded a sensitivity and PPV of 94.2% and 93.7% (see Table 4). The results of regression and correlation analyses performed on the IBrIs estimated from FRG signals are outlined in Table 5, while the results of the Bland–Altman analyses are reported in Table 6. The regression and Bland–Altman plots are shown in Figure 15. The statistical analyses carried out on a total of 2720 IBrIs reported an R^2^ in excess of 0.96, a unit slope, and an intercept of 0.020 s, as well as a non-significant bias and LoAs of [−0.550; 0.536] s.

## 4. Discussion

This study evaluated the performance of a flexible PVDF sensor for FCG by comparing it with a traditional PZT sensor. The results showed that the morphologies of the FCG signals recorded by the two sensors were highly similar, with a median Pearson’s correlation coefficient of 0.96. This finding confirmed the suitability of the proposed PVDF sensor for FCG studies. In addition, the dHF-FCG signals and HS-FCG signals acquired by the PVDF sensor turned out to share very similar morphologies with, respectively, SCG signals obtained from the accelerometer and PCG signals obtained from the electronic stethoscope. It is worth noting that the chest piece and the tubing of the electronic stethoscope influenced its dynamic response, and that, in the experiments, it was not possible to place the stethoscope exactly on the same measurement site of the PVDF/PZT/accelerometer sensors assembly, which also explains the delays observed between PCG signals acquired by the stethoscope and audible HS-FCG signals provided by the PVDF sensor. Nonetheless, the similarity between PCG and HS-FCG signals was fairly high.

This study also evaluated the performance of the Forcecardiography technique in cardio-respiratory monitoring on a larger cohort of subjects with respect to previous studies. The results confirmed the high performance of the PZT sensor reported in [31] for the detection of heartbeats and respiratory acts, and the estimation of inter-beat and inter-breath intervals. The PVDF sensor demonstrated comparable, and sometimes higher, performances with respect to the PZT sensor, which provided further proof of its suitability for accurate cardio-respiratory monitoring.

The flexible PVDF sensor presents several advantages with respect to the PZT sensor. Indeed, the brittle ceramic material of the PZT sensor makes it rigid and prone to cracking, while the flexible materials of the PVDF sensor makes it more robust and adaptable to the curved surfaces of human body, which is appealing for wearable applications. In addition, the PVDF sensor is less prone to thermal drift with respect to the PZT sensor, which yields improved stability under varying temperature conditions. Furthermore, the PVDF is lead-free, while the PZT sensor contains a certain amount of lead [34], which might raise some concerns about safety and biocompatibility for applications involving direct prolonged skin contact.

This study has some limitations. The performance of the flexible PVDF sensor was tested only on a cohort of healthy subjects at rest. A more extensive assessment should be carried out on a larger cohort including pathological subjects, possibly performing physical activities, in order to confirm the promising performance of the PVDF sensor in continuous, long-term, cardio-respiratory monitoring during activities of daily living. For practical reasons, it was not feasible to acquire signals via the PVDF sensor and the electronic stethoscope from the same site on the chest. This undoubtedly prevented a comparison of signals originating from the same mechanical vibration, as it is known that heart sounds captured from different sites on the chest exhibit different morphologies [59]. The long-term robustness of the PVDF FCG sensor to mechanical stress, and to contact with body fluids and chemical agents was not determined. Such an assessment should be carried out to verify the practical suitability of the sensor for long-term monitoring.

Future studies could investigate the integration of the flexible PVDF sensor in garments or wristbands to assess its performance in daily life scenarios. Moreover, the development of a PVDF transducers matrix on a single flexible support layer could enable the investigation of mechanical vibration maps over the chest to gain additional insights into the comprehension of cardiac mechanics. Further works should focus on performing extensive testing of the PVDF FCG sensor on a wider population, including pathological subjects, also during physical activities or stress tests, to confirm its suitability for cardio-respiratory monitoring during daily life activities and, possibly, in sport performances.

## 5. Conclusions

This study presented a flexible PVDF sensor for FCG measurements and assessed its performances. The flexible PVDF sensor acquired FCG signals with high similarity to those provided by a PZT sensor used in previous FCG studies. The PVDF FCG sensor also demonstrated comparable or higher performances in the detection of heartbeats and respiratory acts, as well as in the estimation of inter-beat and inter-breath intervals on a cohort of 35 healthy subjects at rest. The flexible PVDF sensor provides several advantages over the PZT sensor, such as higher robustness and conformability to the human body, as well as improved stability to thermal drift. All these features make the flexible PVDF sensor more suitable for integration into wearable devices for continuous, long-term cardio-respiratory monitoring of subjects.

## Figures and Tables

**Table 1:** The overall results of statistical analysis in heartbeat detection for PVDF and PZT sensors.

Sensor	Signal	Sensitivity (%)	PPV (%)
PVDF	dHF-FCG	92.9	96.9
	HS-FCG	77.7	86.8
PZT	dHF-FCG	93.2	98.2
	HS-FCG	75.0	88.8

**Table 2:** The overall results of correlation and linear regression analysis on inter-beat intervals obtained from ECG and FCG signals provided by PVDF and PZT sensor. The table also reports the confidence intervals of slope and intercept.

Sensor	Signal	N_IBIs_	R^2^	Slope	CI_slope_	Intercept (ms)	CI_intercept_ (ms)
PVDF	dHF-FCG	13,412	>0.99	1.001	[1.001; 1.002]	−0.938	[−1.271; −0.606]
	HS-FCG	10,023	>0.99	1.002	[1.001; 1.002]	−1.273	[−1.629; −0.917]
PZT	dHF-FCG	13,495	>0.99	1.002	[1.002; 1.003]	−1.847	[−2.215; −1.479]
	HS-FCG	9519	>0.99	1.002	[1.002; 1.003]	−1.743	[−2.130; −1.359]

**Table 3:** The overall results of Bland–Altman analysis on inter-beat intervals obtained from ECG and FCG signals provided by PVDF and PZT sensor. The table also reports the confidence intervals of bias and limit of agreement. Since the inter-beat intervals measurement differences were distributed with a non-normal distribution, the bias was estimated as the median of differences and the limits of agreement as the 2.5th and 97.5th percentiles, respectively.

Sensor	Signal	N_IBIs_	Bias (ms)	CI_bias_ (ms)	LoA (ms)	CI_LoA min_ (ms)	CI_LoA max_ (ms)
PVDF	dHF-FCG	13,412	0.00	[0.00; 0.00]	[−5.300; 5.000]	[−5.500; −5.100]	[4.800; 5.100]
	HS-FCG	10,023	0.00	[0.00; 0.00]	[−4.200; 4.400]	[−4.400; −4.000]	[4.200; 4.600]
PZT	dHF-FCG	13,495	0.00	[0.00; 0.10]	[−6.100; 5.700]	[−6.400; −5.900]	[5.500; 6.000]
	HS-FCG	9519	0.00	[0.00; 0.00]	[−4.800; 4.200]	[−5.200; −4.600]	[4.000; 4.500]

**Table 4:** Overall results of statistical analysis in respiratory acts detection for PVDF and PZT sensors.

Signal	Sensor	Sensitivity (%)	PPV (%)
FRG	PVDF	95.1	92.5
	PZT	94.2	93.7

**Table 5:** The overall results of correlation and linear regression analysis on inter-breath intervals obtained from ERB and FRG signals provided by PVDF and PZT sensor. The table also reports the confidence intervals of slope and intercept.

Signal	Sensor	N_IBrIs_	R^2^	Slope	CI_slope_	Intercept (s)	CI_intercept_ (s)
FRG	PVDF	2752	0.967	0.994	[0.987; 1.00]	0.019	[−0.007; 0.044]
	PZT	2720	0.963	0.993	[0.986; 1.00]	0.020	[−0.008; 0.048]

**Table 6:** The overall results of Bland–Altman analysis on inter-breath intervals obtained from ERB and FRG signals provided by PVDF and PZT sensors. The table also reports the confidence intervals of bias and limit of agreement. Since the inter-breath intervals measurement differences was distributed with a non-normal distribution, the bias was estimated as the median of differences and the limits of agreement as the 2.5th and 97.5th percentiles, respectively.

Signal	Sensor	N_IBrIs_	Bias (s)	CI_bias_ (s)	LoA (s)	CI_LoA min_ (s)	CI_LoA max_ (s)
FRG	PVDF	2752	−0.002	[−0.009; 0.001]	[−0.510;0.513]	[−0.572; −0.454]	[0.465; 0.591]
	PZT	2720	−0.003	[−0.008; 0.002]	[−0.550; 0.536]	[−0.602; −0.496]	[0.477; 0.607]

## Data Availability

The datasets presented in this article are not readily available because informed consent from the subjects involved was obtained only for this study and not for public availability. Requests to access the datasets should be directed to E.A. (emilio.andreozzi@unina.it).

## Supplementary Materials

The following supporting information can be downloaded at https://www.mdpi.com/article/10.3390/s25051608/s1. Table S1: “Cross-correlation indices of PVDF FCG sensor signal vs. PZT FCG sensor signal, HF-FCG vs. SCG and HS-FCG vs. PCG”. Table S2: “The number of R-peaks and TP heartbeats identified per subject in the ECG and dHF-FCG signals (extracted from PVDF sensor signal), together with FPs and FNs and the number of compared inter-beat intervals (IBIs)”. Table S3: “The number of R-peaks and TP heartbeats identified per subject in the ECG and HS-FCG signals (extracted from PVDF sensor signal), together with FPs and FNs and the number of compared inter-beat intervals (IBIs)”. Table S4: “The number of R-peaks and TP heartbeats identified per subject in the ECG and dHF-FCG signals (extracted from PZT sensor signal), together with FPs and FNs and the number of compared inter-beat intervals (IBIs)”. Table S5: “The number of R-peaks and TP heartbeats identified per subject in the ECG and HS-FCG signals (extracted from PZT sensor signal), together with FPs and FNs and the number of compared inter-beat intervals (IBIs)”. Table S6: “The number of respiratory acts accurately identified (TP) per subject in the FRG (extracted from the PVDF sensor signal) and ERB reference signals, together with FPs and FNs and the number of compared inter-breath intervals (IBrIs)”. Table S7: “The number of respiratory acts accurately identified (TP) per subject in the FRG (extracted from the PZT sensor signal) and ERB reference signals, together with FPs and FNs and the number of compared inter-breath intervals (IBrIs)”.

## References

[1] Wells B. (1954). Phonocardiography. Br. Med. J..

[2] Rappaport M.B., Sprague H.B. (1942). The Graphic Registration of the Normal Heart Sounds. Am. Heart J..

[3] Ismail S., Siddiqi I., Akram U. (2018). Localization and Classification of Heart Beats in Phonocardiography Signals—A Comprehensive Review. EURASIP J. Adv. Signal Process..

[4] Gordon J.W. (1877). Certain Molar Movements of the Human Body Produced by the Circulation of the Blood. J. Anat. Physiol..

[5] Burger H.C., Noordergraaf A. (1956). Physical Basis of Ballistocardiography. III. Am. Heart J..

[6] Deuchar D.C. (1967). Ballistocardiography. Br. Heart J..

[7] Starr I. (1958). The Relation of the Ballistocardiogram to Cardiac Function. Am. J. Cardiol..

[8] Knoop A.A. (1965). Experimental Investigations on Ultra-Low Frequency Displacement Ballistocardiography <Experimentele Onderzoekingen over de Ultra-Laagfrequente Verplaatsingsballisto-Cardiografie>.

[9] Sadek I., Biswas J., Abdulrazak B. (2019). Ballistocardiogram Signal Processing: A Review. Health Inf. Sci. Syst..

[10] Vogt E., MacQuarrie D., Neary J.P. (2012). Using Ballistocardiography to Measure Cardiac Performance: A Brief Review of Its History and Future Significance. Clin. Physiol. Funct. Imaging.

[11] Marey E.-J., Masson G. (1885). La Méthode Graphique Dans Les Sciences Expérimentales et Principalement en Physiologie et en Médecine.

[12] Benchimol A., Dimond E.G. (1962). The Apex Cardiogram in Ischaemic Heart Disease. Br. Heart J..

[13] Gupta M.C., Mathur K.S. (1968). Apexcardiogram. Indian Heart J..

[14] Babskiĭ E.B., Karpman V.L. (1964). Dynamocardiography.

[15] Eddleman E.E., Willis K., Reeves T.J., Harrison T.R. (1953). The Kinetocardiogram. Circulation.

[16] Salerno D.M. (1990). Seismocardiography: A New Technique for Recording Cardiac Vibrations. Concept, Method, and Initial Observations. J. Cardiovasc. Technol..

[17] Zanetti J.M., Tavakolian K. Seismocardiography: Past, Present and Future. Proceedings of the 2013 35th Annual International Conference of the IEEE Engineering in Medicine and Biology Society (EMBC).

[18] Inan O.T., Migeotte P.-F., Park K.-S., Etemadi M., Tavakolian K., Casanella R., Zanetti J., Tank J., Funtova I., Prisk G.K. (2015). Ballistocardiography and Seismocardiography: A Review of Recent Advances. IEEE J. Biomed. Health Inf..

[19] Taebi A., Solar B.E., Bomar A.J., Sandler R.H., Mansy H.A. (2019). Recent Advances in Seismocardiography. Vibration.

[20] Castiglioni P., Faini A., Parati G., Di Rienzo M. (2007). Wearable Seismocardiography. Annu. Int. Conf. IEEE Eng. Med. Biol. Soc..

[21] Etemadi M., Inan O.T. (2018). Wearable Ballistocardiogram and Seismocardiogram Systems for Health and Performance. J. Appl. Physiol..

[22] Jafari Tadi M., Lehtonen E., Saraste A., Tuominen J., Koskinen J., Teräs M., Airaksinen J., Pänkäälä M., Koivisto T. (2017). Gyrocardiography: A New Non-Invasive Monitoring Method for the Assessment of Cardiac Mechanics and the Estimation of Hemodynamic Variables. Sci. Rep..

[23] Sieciński S., Kostka P.S., Tkacz E.J. (2020). Gyrocardiography: A Review of the Definition, History, Waveform Description, and Applications. Sensors.

[24] Yang C., Tavassolian N. (2018). Combined Seismo- and Gyro-Cardiography: A More Comprehensive Evaluation of Heart-Induced Chest Vibrations. IEEE J. Biomed. Health Inform..

[25] Shandhi M.M.H., Semiz B., Hersek S., Goller N., Ayazi F., Inan O.T. (2019). Performance Analysis of Gyroscope and Accelerometer Sensors for Seismocardiography-Based Wearable Pre-Ejection Period Estimation. IEEE J. Biomed. Health Inf..

[26] Yang C., Tang S., Tavassolian N. (2017). Utilizing Gyroscopes Towards the Automatic Annotation of Seismocardiograms. IEEE Sens. J..

[27] D’Mello Y., Skoric J., Xu S., Roche P.J.R., Lortie M., Gagnon S., Plant D.V. (2019). Real-Time Cardiac Beat Detection and Heart Rate Monitoring from Combined Seismocardiography and Gyrocardiography. Sensors.

[28] Hossein A., Rabineau J., Gorlier D., Del Rio J.I.J., van de Borne P., Migeotte P.-F., Nonclercq A. (2021). Kinocardiography Derived from Ballistocardiography and Seismocardiography Shows High Repeatability in Healthy Subjects. Sensors.

[29] Hossein A., Rabineau J., Gorlier D., Pinki F., van de Borne P., Nonclercq A., Migeotte P.-F. (2021). Effects of Acquisition Device, Sampling Rate, and Record Length on Kinocardiography during Position-Induced Haemodynamic Changes. Biomed. Eng. Online.

[30] Jafari Tadi M., Koivisto T., Pänkäälä M., Paasio A. (2014). Accelerometer-Based Method for Extracting Respiratory and Cardiac Gating Information for Dual Gating during Nuclear Medicine Imaging. Int. J. Biomed. Imaging.

[31] Andreozzi E., Gargiulo G.D., Esposito D., Bifulco P. (2021). A Novel Broadband Forcecardiography Sensor for Simultaneous Monitoring of Respiration, Infrasonic Cardiac Vibrations and Heart Sounds. Front. Physiol..

[32] Andreozzi E., Centracchio J., Punzo V., Esposito D., Polley C., Gargiulo G.D., Bifulco P. (2021). Respiration Monitoring via Forcecardiography Sensors. Sensors.

[33] Centracchio J., Andreozzi E., Esposito D., Gargiulo G.D., Bifulco P. (2022). Detection of Aortic Valve Opening and Estimation of Pre-Ejection Period in Forcecardiography Recordings. Bioengineering.

[34] Aksel E., Jones J.L. (2010). Advances in Lead-Free Piezoelectric Materials for Sensors and Actuators. Sensors.

[35] Rasheed A., Iranmanesh E., Li W., Ou H., Andrenko A.S., Wang K. (2017). A Wearable Autonomous Heart Rate Sensor Based on Piezoelectric-Charge-Gated Thin-Film Transistor for Continuous Multi-Point Monitoring. Annu. Int. Conf. IEEE Eng. Med. Biol. Soc..

[36] Parlato S., Esposito D., Centracchio J., Andreozzi E., Gragnaniello M., Riccio M., Bifulco P. (2024). A New, Simple Wrist-Mounted PVDF Sensor for Continuous Heart Rate Monitoring. Proceedings of the 2024 IEEE Sensors Applications Symposium (SAS).

[37] Choi S., Jiang Z. (2006). A Novel Wearable Sensor Device with Conductive Fabric and PVDF Film for Monitoring Cardiorespiratory Signals. Sens. Actuators A Phys..

[38] Maity K., Garain S., Henkel K., Schmeißer D., Mandal D. (2020). Self-Powered Human-Health Monitoring through Aligned PVDF Nanofibers Interfaced Skin-Interactive Piezoelectric Sensor. ACS Appl. Polym. Mater..

[39] Hu Y., Kang W., Fang Y., Xie L., Qiu L., Jin T. (2018). Piezoelectric Poly(Vinylidene Fluoride) (PVDF) Polymer-Based Sensor for Wrist Motion Signal Detection. Appl. Sci..

[40] Xin Y., Qi X., Qian C., Tian H., Ling Z., Jiang Z. (2014). A Wearable Respiration and Pulse Monitoring System Based on PVDF Piezoelectric Film. Integr. Ferroelectr..

[41] Li S., Zhou T., Liu M., Zhao Q., Liu Y. (2024). An Intelligent Non-Invasive Blood Pressure Monitoring System Based on a Novel Polyvinylidene Fluoride Piezoelectric Thin Film. Micromachines.

[42] Zaszczyńska A., Gradys A., Sajkiewicz P. (2020). Progress in the Applications of Smart Piezoelectric Materials for Medical Devices. Polymers.

[43] Jiang Y., Hamada H., Shiono S., Kanda K., Fujita T., Higuchi K., Maenaka K. (2010). A PVDF-Based Flexible Cardiorespiratory Sensor with Independently Optimized Sensitivity to Heartbeat and Respiration. Procedia Eng..

[44] Xu T., Jin L., Ao Y., Zhang J., Sun Y., Wang S., Qu Y., Huang L., Yang T., Deng W. (2024). All-Polymer Piezo-Ionic-Electric Electronics. Nat. Commun..

[45] Ha T., Tran J., Liu S., Jang H., Jeong H., Mitbander R., Huh H., Qiu Y., Duong J., Wang R.L. (2019). A Chest-Laminated Ultrathin and Stretchable E-Tattoo for the Measurement of Electrocardiogram, Seismocardiogram, and Cardiac Time Intervals. Adv. Sci..

[46] Jayarathna T., Gargiulo G.D., Breen P.P. (2020). Continuous Vital Monitoring During Sleep and Light Activity Using Carbon-Black Elastomer Sensors. Sensors.

[47] Pan J., Tompkins W.J. (1985). A Real-Time QRS Detection Algorithm. IEEE Trans. Biomed. Eng..

[48] Sedghamiz H. (2018). BioSigKit: A Matlab Toolbox and Interface for Analysis of BioSignals. J. Open Source Softw..

[49] Savitzky A., Golay M.J.E. (1964). Smoothing and Differentiation of Data by Simplified Least Squares Procedures. Anal. Chem..

[50] Centracchio J., Parlato S., Esposito D., Andreozzi E. (2024). Accurate Localization of First and Second Heart Sounds via Template Matching in Forcecardiography Signals. Sensors.

[51] Centracchio J., Muto V. Heartbeats Localization in Forcecardiography Signals Via Template Matching. Proceedings of the 2022 E-Health and Bioengineering Conference (EHB).

[52] Centracchio J., Parlato S., Esposito D., Bifulco P., Andreozzi E. (2023). ECG-Free Heartbeat Detection in Seismocardiography Signals via Template Matching. Sensors.

[53] Parlato S., Centracchio J., Esposito D., Bifulco P., Andreozzi E. (2023). Heartbeat Detection in Gyrocardiography Signals without Concurrent ECG Tracings. Sensors.

[54] Parlato S., Centracchio J., Esposito D., Bifulco P., Andreozzi E. (2023). ECG-Free Heartbeat Detection in Seismocardiography and Gyrocardiography Signals Provides Acceptable Heart Rate Variability Indices in Healthy and Pathological Subjects. Sensors.

[55] Parlato S., Muto V., Bifulco P., Costin H.-N., Magjarević R., Petroiu G.G. (2024). Accurate ECG-Free Heartbeats Localization in Long-Lasting SCG Recordings. Proceedings of the Advances in Digital Health and Medical Bioengineering.

[56] Altman D.G., Bland J.M. (1983). Measurement in Medicine: The Analysis of Method Comparison Studies. J. R. Stat. Soc. Ser. D Stat..

[57] Giavarina D. (2015). Understanding Bland Altman Analysis. Biochem. Med..

[58] Klein R. Bland-Altman and Correlation Plot. https://it.mathworks.com/matlabcentral/fileexchange/45049-bland-altman-and-correlation-plot.

[59] Guo B., Tang H., Xia S., Wang M., Hu Y., Zhao Z. (2022). Development of a Multi-Channel Wearable Heart Sound Visualization System. J. Pers. Med..

