# Supplementary material for: A Flexible PVDF Sensor for Forcecardiography
Source: Sensors (Basel). 2025 Mar 6;25(5):1608. doi: 10.3390/s25051608 (PMC11902622; doi:10.3390/s25051608)
Supplement: Supplementary file 1 [file sensors-25-01608-s001.zip › sensors-3504695-supplementary.pdf]

Supplementary Material

A Flexible PVDF Sensor for Forcecardiography

Salvatore Parlato [1], Jessica Centracchio [1,*], Eliana Cinotti [1], Gaetano D. Gargiulo [2], Daniele Esposito [3], Paolo Bifulco [1,*] and Emilio Andreozzi [1]

[1] Department of Electrical Engineering and Information Technologies, University of Naples Federico II, Via Claudio, 21, 80125 Naples, Italy; salvatore.parlato@unina.it (S.P.); eliana.cinotti@unina.it (E.C.); emilio.andreozzi@unina.it (E.A.)

[2] School of Engineering, Design and Built Environment, Western Sydney University, Penrith, NSW 2751, Australia; g.gargiulo@westernsydney.edu.au

[3] Department of Information and Electrical Engineering and Applied Mathematics, University of Salerno, Via Giovanni Paolo II, 132, 84084 Fisciano, Italy; daesposito@unisa.it

* Correspondence: jessica.centracchio@unina.it (J.C.); paolo.bifulco@unina.it (P.B.)

Table S1. Cross-correlation indices of PVDF FCG sensor signal vs. PZT FCG sensor signal, HF-FCG vs. SCG and HS-FCG vs. PCG.

Subject #ID	PVDF vs. PZT	dHF-FCG vs. SCG	HS-FCG vs. PCG
1	0.92	0.91	0.83
2	0.98	0.82	0.85
3	0.99	0.81	0.83
4	0.98	0.85	0.87
5	0.99	0.85	0.68
6	0.96	0.91	0.85
7	>0.99	0.77	0.89
8	0.98	0.80	0.61
9	>0.99	0.81	0.62
10	0.86	0.81	0.80
11	0.91	0.87	0.74
12	0.93	0.92	0.81
13	0.93	0.97	0.73
14	0.97	0.81	0.74
15	0.89	0.73	0.80
16	0.98	0.77	0.84
17	0.96	0.70	0.81
18	0.98	0.88	0.89
19	0.95	0.95	0.76
20	>0.99	0.87	0.74
21	0.79	0.88	0.81
22	>0.99	0.93	0.81
23	0.99	0.86	0.61
24	0.90	0.89	0.70
25	0.86	0.90	0.77
26	0.94	0.90	0.79
27	0.93	0.77	0.62
28	0.90	0.76	0.78
29	0.96	0.95	0.69
30	0.91	0.73	0.88
31	0.93	0.85	0.76
32	0.93	0.83	0.95
33	0.97	0.85	0.80
34	0.98	0.79	0.83
35	0.97	0.83	0.75

Table S2. The number of R-peaks and TP heartbeats identified per subject in the ECG and dHF-FCG signals (extracted from PVDF sensor signal), together with FPs and FNs and the number of compared inter-beat intervals (IBIs).

Subject #ID	R-peaks	TP	FP	FN	Compared IBIs
1	437	414	2	23	394
2	414	402	5	12	389
3	367	353	13	14	337
4	376	339	8	37	308
5	494	463	16	31	448
6	387	229	152	158	158
7	421	294	14	127	216
8	433	328	19	105	276
9	381	369	12	12	356
10	393	331	45	62	281
11	385	379	5	6	372
12	363	363	0	0	362
13	390	390	0	0	389
14	539	462	26	77	410
15	564	527	0	37	496
16	429	395	7	34	373
17	629	599	12	30	577
18	387	381	6	6	373
19	490	490	0	0	489
20	360	360	7	0	358
21	391	391	0	0	390
22	371	363	7	8	355
23	418	418	0	0	417
24	425	378	17	47	339
25	444	444	0	0	443
26	602	594	4	8	588
27	569	539	15	30	510
28	436	329	49	107	267
29	453	440	4	13	429
30	399	319	3	80	246
31	389	384	3	5	377
32	389	389	0	0	388
33	429	418	5	11	406
34	449	449	0	0	448
35	448	448	0	0	447
**TOTAL**	**15251**	**14171**	**456**	**1080**	**13412**

Table S3. The number of R-peaks and TP heartbeats identified per subject in the ECG and HS-FCG signals (extracted from PVDF sensor signal), together with FPs and FNs and the number of compared inter-beat intervals (IBIs).

Subject #ID	R-peaks	TP	FP	FN	Compared IBIs
1	437	309	59	128	249
2	414	406	6	8	397
3	367	192	172	175	83
4	376	305	69	71	269
5	494	482	1	12	470
6	387	211	172	176	127
7	421	212	145	209	146
8	433	307	3	126	249
9	381	273	36	108	220
10	393	268	99	125	205
11	385	269	163	116	187
12	363	317	47	46	274
13	390	387	3	3	382
14	539	413	10	126	359
15	564	492	23	72	446
16	429	334	1	95	279
17	629	542	58	87	492
18	387	381	6	6	373
19	490	488	1	2	485
20	360	274	78	86	242
21	391	241	136	150	127
22	371	313	56	58	269
23	418	276	9	142	215
24	425	293	42	132	232
25	444	349	62	95	300
26	602	403	37	199	296
27	569	326	61	243	221
28	436	335	24	101	268
29	453	405	18	48	378
30	399	322	23	77	251
31	389	222	56	167	143
32	389	389	0	0	388
33	429	288	114	141	221
34	449	438	3	11	426
35	448	389	17	59	354
**TOTAL**	**15251**	**11851**	**1810**	**3400**	**10023**

Table S4. The number of R-peaks and TP heartbeats identified per subject in the ECG and dHF-FCG signals (extracted from PZT sensor signal), together with FPs and FNs and the number of compared inter-beat intervals (IBIs).

Subject #ID	R-peaks	TP	FP	FN	Compared IBIs
1	437	431	2	6	426
2	414	270	8	144	199
3	367	338	26	29	307
4	376	362	5	14	348
5	494	450	18	44	423
6	387	282	9	105	229
7	421	394	1	27	369
8	433	427	2	6	419
9	381	348	31	33	319
10	393	344	7	49	299
11	385	382	0	3	377
12	363	353	10	10	348
13	390	387	1	3	382
14	539	538	0	1	535
15	564	557	1	7	551
16	429	402	8	27	385
17	629	615	0	14	604
18	387	387	0	0	386
19	490	490	0	0	489
20	360	356	11	4	350
21	391	372	2	19	353
22	371	369	2	2	365
23	418	418	0	0	417
24	425	326	47	99	254
25	444	438	0	6	430
26	602	600	0	2	596
27	569	553	8	16	535
28	436	258	27	178	188
29	453	409	3	44	388
30	399	311	11	88	233
31	389	379	1	10	368
32	389	384	5	5	378
33	429	429	0	0	428
34	449	407	17	42	379
35	448	443	3	5	438
**TOTAL**	**15251**	**14209**	**266**	**1042**	**13495**

Table S5. The number of R-peaks and TP heartbeats identified per subject in the ECG and HS-FCG signals (extracted from PZT sensor signal), together with FPs and FNs and the number of compared inter-beat intervals (IBIs).

Subject #ID	R-peaks	TP	FP	FN	Compared IBIs
1	437	218	11	219	136
2	414	410	2	4	404
3	367	254	43	113	167
4	376	298	47	78	245
5	494	388	80	106	349
6	387	242	128	145	164
7	421	224	139	197	162
8	433	287	23	146	230
9	381	269	72	112	204
10	393	245	76	148	137
11	385	270	95	115	230
12	363	363	0	0	362
13	390	386	0	4	381
14	539	450	0	89	408
15	564	523	7	41	469
16	429	382	12	47	349
17	629	619	0	10	611
18	387	294	93	93	245
19	490	453	7	37	426
20	360	159	28	201	93
21	391	189	115	202	89
22	371	317	37	54	272
23	418	255	0	163	197
24	425	327	46	98	253
25	444	440	4	4	434
26	602	403	3	199	286
27	569	272	4	297	155
28	436	252	78	184	181
29	453	355	6	98	319
30	399	313	72	86	253
31	389	192	57	197	99
32	389	389	0	0	388
33	429	310	72	119	222
34	449	409	12	40	377
35	448	282	67	166	222
**TOTAL**	**15251**	**11439**	**1436**	**3812**	**9519**

Table S6. The number of respiratory acts accurately identified (TP) per subject in the FRG (extracted from the PVDF sensor signal) and ERB reference signals, together with FPs and FNs and the number of compared inter-breath intervals (IBrIs).

Subject #ID	Reference respiratory acts	TP	FP	FN	Compared IBrIs
1	87	84	2	3	78
2	71	70	0	1	69
3	113	111	1	2	109
4	58	58	0	0	57
5	121	121	0	0	120
6	60	46	16	14	39
7	55	55	1	0	52
8	45	43	5	2	39
9	64	63	0	1	61
10	80	76	4	4	71
11	124	124	4	0	122
12	58	58	0	0	57
13	92	87	2	5	81
14	67	52	16	15	45
15	113	103	9	10	93
16	72	69	21	3	65
17	62	59	14	3	45
18	90	90	0	0	89
19	140	138	3	2	135
20	99	93	11	6	89
21	114	112	2	2	109
22	94	94	0	0	93
23	56	46	13	10	40
24	70	67	12	3	64
25	122	122	1	0	121
26	130	126	5	4	121
27	159	155	8	4	153
28	56	38	18	18	30
29	99	78	19	21	66
30	82	82	1	0	81
31	66	53	31	13	43
32	56	56	0	0	55
33	164	164	1	0	163
34	64	60	16	4	56
35	42	42	0	0	41
**TOTAL**	**3045**	**2895**	**236**	**150**	**2752**

Table S7. The number of respiratory acts accurately identified (TP) per subject in the FRG (extracted from the PZT sensor signal) and ERB reference signals, together with FPs and FNs and the number of compared inter-breath intervals (IBrIs).

Subject #ID	Reference respiratory acts	TP	FP	FN	Compared IBrIs
1	87	75	14	12	65
2	71	71	0	0	70
3	113	113	0	0	112
4	58	57	2	1	55
5	121	85	16	36	64
6	60	46	18	14	34
7	55	53	6	3	50
8	45	43	3	2	40
9	64	63	1	1	61
10	80	80	0	0	79
11	124	120	4	4	116
12	58	58	0	0	57
13	92	88	3	4	83
14	67	67	1	0	66
15	113	113	1	0	112
16	72	70	4	2	68
17	62	37	46	25	26
18	90	90	0	0	89
19	140	135	4	5	130
20	99	98	5	1	96
21	114	114	0	0	113
22	94	94	0	0	93
23	56	55	2	1	53
24	70	50	23	20	38
25	122	122	1	0	121
26	130	118	8	12	109
27	159	144	10	15	134
28	56	55	0	1	53
29	99	87	17	12	78
30	82	82	0	0	81
31	66	65	0	1	63
32	56	56	0	0	55
33	164	161	0	3	158
34	64	61	5	3	57
35	42	42	1	0	41
**TOTAL**	**3045**	**2868**	**195**	**178**	**2720**
